# Controlled Cellular Delivery of Amphiphilic Cargo by Redox‐Responsive Nanocontainers

**DOI:** 10.1002/advs.201901935

**Published:** 2019-10-24

**Authors:** Wilke C. de Vries, Sergej Kudruk, David Grill, Maximilian Niehues, Anna Livia Linard Matos, Maren Wissing, Armido Studer, Volker Gerke, Bart Jan Ravoo

**Affiliations:** ^1^ Center for Soft Nanoscience and Organic Chemistry Institute Westfälische Wilhelms‐Universität Münster Busso‐Peus‐Str. 10 Münster 48149 Germany; ^2^ Institute of Medical Biochemistry Center for Molecular Biology of Inflammation Westfälische Wilhelms‐Universität Münster Von‐Esmarch‐Str. 56 Münster 48149 Germany

**Keywords:** disulfides, intracellular delivery, lipids, liposomes, polymers, self‐assembly

## Abstract

The specific transport of amphiphilic compounds such as fluorescently labeled phospholipids into cells is a prerequisite for the analysis of highly dynamic cellular processes involving these molecules, e.g., the intracellular distribution and metabolism of phospholipids. However, cellular delivery remains a challenge as it should not affect the physiological integrity and morphology of the cell membrane. To address this, polymer nanocontainers based on redox‐responsive cyclodextrin (CD) amphiphiles are prepared, and their potential to deliver fluorescently labeled phospholipids to intracellular membrane compartments is analyzed. It is shown that mixtures of reductively degradable cyclodextrin amphiphiles and different phospholipids form liposome‐like vesicles (CD–lipid vesicles, C_SS_LV) with a homogeneous distribution of each lipid. Host–guest‐mediated self‐assembly of a cystamine‐crosslinked polymer shell on these C_SS_LV produces polymer‐shelled liposomal vesicles (P_SS_C_SS_LV) with the unique feature of a redox‐sensitive C_SS_LV core and reductively degradable polymer shell. P_SS_C_SS_LV show high stability and a redox‐sensitive release of the amphiphilic cargo. Live cell experiments reveal that the novel P_SS_C_SS_LV are readily internalized by primary human endothelial cells and that the reductive microenvironment of the cells' endosomes triggers the release of the amphiphilic cargo into the cytosol. Thus, P_SS_C_SS_LV represent a highly efficient system to transport lipid‐like amphiphilic cargo into the intracellular environment.

## Graphical abstract

Novel polymer nanocontainers based on redox‐responsive cyclodextrin amphiphiles are introduced. Mixed with phospholipids, these amphiphiles form liposome‐like vesicles that can be encapsulated in a cystamine‐crosslinked polymer shell producing polymer‐shelled liposomal vesicles with a redox‐sensitive core and reductively degradable polymer shell. Live cell imaging shows that these nanocontainers can deliver amphiphilic cargo, specifically labeled phospholipids into human cells.

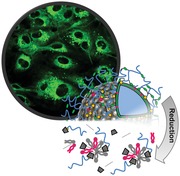

The transport of fluorescently labeled phospholipids into the cell's interior can permit a live cell analysis of dynamic membrane‐related processes involving such lipids that include lipid transport, lipid microdomain formation, or the interaction of lipids with membrane proteins.^1^, ^2^, ^3^, ^4^, ^5^, ^6^, ^7^, ^8^, ^9^, ^10^, ^11^, ^12^, ^13^, ^14^, ^15^, ^16^ An important prerequisite for such studies is a mode of intracellular delivery that does not impair the physiological integrity of the cell membrane. Fusogenic liposomes have been introduced to integrate amphiphilic lipids into the plasma membrane; however, the presence of fusogenic lipids in these systems can affect membrane properties.^6^ Other approaches used pH‐responsive polymersomes as delivery vehicles.^16^ Such polymersomes, initially reported by Armes and co‐workers, are based on diblock copolymers consisting of a hydrophilic poly(2‐(diisopropylamino)ethyl methacrylate) (PDPA) block and a hydrophobic poly(2‐(methacryloyloxy)ethyl phosphorylcholine) (PMPC) block.^17^ Hydrophilic cargo encapsulated in the polymersomes could be released by protonation of the PDPA blocks and a resulting disassembly most likely occurring in the acidic late‐endosomal/lysosomal cell compartment. A different approach to generate responsive vehicles for amphiphile delivery is based on amphiphilic cyclodextrin (CD) derivatives, in which the hydrophilic cyclodextrin headgroup is connected via a disulfide (_ss_) bridge to hydrophobic alkyl chains of different lengths, thereby introducing a reductively cleavable bond.^18^ Such cyclodextrin vesicles were shown to trap hydrophobic dyes that could be released by reductive degradation. Here, we employed similar CD vesicles as basis for novel redox‐sensitive nanocontainers permitting the efficient transport of amphiphilic phospholipids into cells.

The rationale of our approach is outlined in **Figure** **1**. Cargo lipids and reductively cleavable β‐cyclodextrin amphiphiles (β‐CD_SS_) were co‐assembled into liposome‐like cyclodextrin vesicle templates and decorated by host–guest chemistry with a reductively cleavable polymer shell.

**Figure 1:**
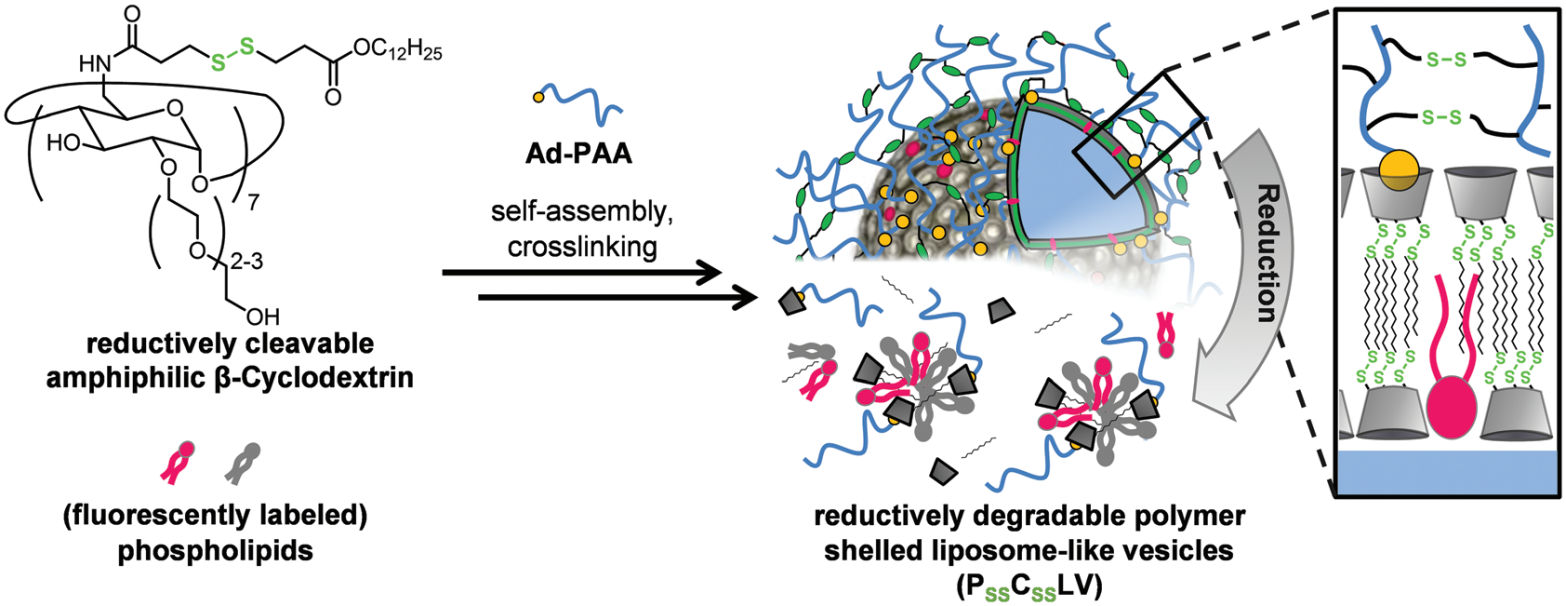
Schematic representation of the assembly and structure of P_SS_C_SS_LV, which entirely dissociate into smaller molecular units by cleavage of the disulfide bonds of both, the cyclodextrin core and the polymer shell.

Reductively cleavable β‐CD_SS_ were synthesized as described^19^ and mixed with different phospholipids to generate redox‐sensitive mixed CD–lipid vesicles (C_SS_LV). Different β‐CD_SS_/lipid mixtures were tested for miscibility and stability, and a ratio of 50 mol% of the reductively cleavable β‐CD_SS_, 25 mol% 1‐palmitoyl‐2‐oleoyl‐*sn*‐glycero‐3‐phospho‐(1′‐rac‐glycerol) (POPG), 20 mol% 1,2‐dioleoyl‐*sn*‐glycero‐3‐phosphate (DOPA), and 5 mol% 1,2‐dioleoyl‐*sn*‐glycero‐3‐phosphocholine (DOPC) was chosen for further experiments. Miscibility was verified by employing β‐CD_SS_/lipid giant unilamellar vesicles (C_SS_L‐GUV). Figures S1–S4 (Supporting Information) show that TopFluor (TF)‐ or nitrobenzoxadiazole (NBD)‐labeled phospholipids (phosphatidylcholine (PC), phosphatidic acid (PA), phosphatidylinositol (PI), phosphatidylinositol‐4,5‐biphosphat (PI(4,5)P_2_)) assume a homogeneous distribution in the mixed β‐CD_SS_/lipid vesicles and that the β‐CD_SS_, which were labeled with adamantane‐terminated rhodamine B (Ad‐TEG‐RhB) by host–guest recognition, are also homogeneously distributed.

The CD/lipid mixtures were then extruded to yield vesicles with an average hydrodynamic diameter of *d*_h_ ≈ 120 nm and a ζ‐potential of ζ ≈ −8 mV that remained stable over several days and also tolerated the incorporation of fluorescently labeled lipid derivatives. In a next step, the C_SS_LV were covered with a disulfide‐crosslinked polymer shell by attaching adamantane‐terminated polyacrylic acid (Ad–PAA) via host–guest interaction.^19^ The formation of homogeneous particles, herein referred to as polymer‐shelled cyclodextrin lipid vesicles (P_SS_C_SS_LV), was assessed by dynamic light scattering (DLS), Fourier‐transform infrared (FT‐IR) spectroscopy, and ζ‐potential measurements. This revealed an increase of the hydrodynamic radius by 25 nm and a decrease in the ζ‐potential from ζ = −7.5 mV to ζ = −19 mV due to deprotonation of acrylic acid moieties of the polymer chain in P_SS_C_SS_LV. The stability of the polymer shell was then increased by 1‐ethyl‐3‐(3‐dimethylaminopropyl)carbodiimide (EDC)‐mediated crosslinking yielding an amide bond with cystamine. During this step, no significant change of the hydrodynamic diameter but an increase of the ζ‐potential to ζ = −12 mV was observed due to the conversion of carboxylic acid to the amide. The successful conversion was also recorded via FT‐IR spectroscopy revealing a decrease of the C=O stretching band of the carboxylic acid and a simultaneous increase of the amide absorption bands (Figure S5, Supporting Information).

P_SS_C_SS_LV contain many disulfide bonds and should disintegrate in a reductive environment. This was analyzed by treatment with the thiol‐based reducing agent dithiothreitol (DTT). Prior to addition of a reducing agent, P_SS_C_SS_LV show an average hydrodynamic diameter of *d*_h_ ≈ 145 nm and appear as circular, flattened objects in transmission electron microscopy (TEM) images (**Figure** **2**), typically observed for vesicles and polymer capsules.^20^, ^21^, ^22^, ^23^ Incubation with DTT led to a change in morphology and a pronounced decrease in size (Figure 2). The reductive disintegration of P_SS_C_SS_LV could be followed by recording the intensity of scattered light as a function of time revealing the colloidal stability of P_SS_C_SS_LV in the absence of DTT and, upon addition of DTT, a pronounced decrease (Figure S6, Supporting Information).

**Figure 2:**
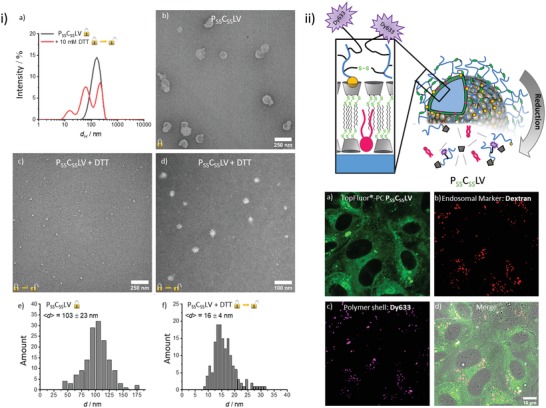
Morphology and cellular delivery of P_SS_C_SS_LV. i) Size change due to reductive disassembly of the nanocontainers. a) Intensity‐weighted size‐distribution of P_SS_C_SS_LV determined by DLS before and after treatment with 10 × 10^−3^ m DTT. A CONTIN‐algorithm for polydisperse samples was used for the analysis. b) TEM images of P_SS_C_SS_LV. c,d) TEM images following reductive cleavage. After 24 h of incubation in 10 × 10^−3^ m DTT, samples were negatively contrasted by 0.5% (w/v) tungstophosphoric acid. e,f) Size distribution of P_SS_C_SS_LV determined by TEM e) before and f) after 24 h incubation in 10 mm DTT. ii) Intracellular delivery of TF–PC incorporated into P_SS_C_SS_LV. Top: Schematic representation of the structure of P_SS_C_SS_LV containing a Dy633‐labeled polymer shell. Bottom: HUVEC were incubated for 2 h with Dy633‐labeled P_SS_C_SS_LV containing TF‐labeled PC and analyzed by confocal microscopy. Images show the uptake of the P_SS_C_SS_LV and the intracellular release of TF–PC. The fluorescent label of the polymer shell, Dy633, is retained in endosomal structures as revealed by labeling with co‐internalized RhB–dextran.

We next assessed whether the reductive disintegration is accompanied by a release of the cargo lipids. Therefore, we performed Förster resonance energy transfer (FRET) experiments employing two populations of P_SS_C_SS_LV, one loaded with rhodamine B‐labeled phosphatidylethanolamine (PE) and another with NBD‐labeled PE. Although no significant decrease in the fluorescence of NBD (λ_em_ = 527 nm) was observed upon mixing with the rhodamine–PE labeled P_SS_C_SS_LV in the absence of a reducing agent, addition of DTT led to a decrease in NBD fluorescence and a concomitant increase in the fluorescence of rhodamine B, suggesting FRET due to close proximity (distance < 10 nm) of the two fluorophore‐labeled lipids (Figure S6 and kinetics in Figure S7, Supporting Information). Most likely, disintegration of P_SS_C_SS_LV had resulted in a dynamic distribution and thus mixing of the cargo lipids within the smaller aggregates identified by DLS and TEM (Figure 2). To verify the specific redox sensitivity of the nanocontainers, we performed control experiments with polymer‐shelled cyclodextrin liposomal vesicle (PCLV) devoid of reductively cleavable disulfide bonds. Therefore, the β‐CD_SS_ were replaced by redox‐stable cyclodextrin amphiphiles,^24^, ^25^ and 2,2′‐(ethylendioxy)bis(ethyleneamine) was used for crosslinking of the polymer shell.^26^ These PCLV remained stable under reducing conditions, i.e., addition of DTT did not result in a change of size distributions or an altered FRET (Figure S6, Supporting Information) verifying that disintegration of P_SS_C_SS_LV is caused by reduction of the disulfide bonds. In another set of controls, we assessed whether a cleavage of the polymer shell alone might already facilitate a release of cargo lipids. Therefore, P_SS_CLV containing only a reductively cleavable polymer shell but a redox‐stable CD vesicle template were synthesized. Figure S6 (Supporting Information) shows that P_SS_CLV behave like PCLV, indicating that a release of cargo lipids requires both, a cleavable polymer shell and a reductively degradable vesicle template.

PC‐derivatives represent the main lipid species in mammalian membranes^27^ and thus were chosen as the first cargo for P_SS_C_SS_LV‐mediated cellular delivery. P_SS_C_SS_LV containing PC labeled with NBD or TF in the alkyl chain were administered to the medium of primary human umbilical vein endothelial cells (HUVEC), and the uptake and intracellular fate of the phospholipid were visualized by confocal fluorescence microscopy. Figure 2 shows that 2 h after addition of the NBD–PC carrying P_SS_C_SS_LV, the NBD label was distributed throughout the cytosol showing a somewhat reticular pattern with a minor enrichment in the perinuclear region. A similar uptake and distribution pattern, showing increased cytosolic delivery with time, was observed for TF–PC (Figure S8, Supporting Information). The distribution of NBD‐ or TF‐labeled PC after P_SS_C_SS_LV‐mediated delivery into HUVEC looked reminiscent of the endoplasmic reticulum (ER) and co‐staining with an ER‐marker indeed revealed considerable co‐localization (Figure S9, Supporting Information).

We also analyzed the intracellular fate of the P_SS_C_SS_LV polymer shell after delivery into HUVEC. Therefore, an amine‐functionalized fluorophore, Dy633, was attached during the crosslinking process by covalent linkage to acrylic acid moieties of the polymer shell. After internalization of this modified P_SS_C_SS_LV, the polymer shell label did not distribute throughout the cytoplasm but appeared in punctate structures that were identified as endosomes by simultaneous uptake of rhodamine B (RhB) conjugated dextran (RhB–Dextran, *M_n_* ≈ 70 kDa) (Figure 2). Together, these results suggest that the P_SS_C_SS_LV are first internalized into the endosomal system before the reductive environment of endosomes triggers disintegration of the redox‐sensitive polymer shell and β‐CD_SS_ core. The degraded polymer shell remains trapped in endosomes whereas the amphiphilic cargo, i.e., the labeled phospholipids, is released from the disintegrated P_SS_C_SS_LV and escapes from endosomes to partition into ER membranes. The enrichment in the ER probably reflects the ubiquitous nature of ER membranes that comprise the majority of intracellular membranes.

To verify the regulatory role of the two disulfide crosslinks and show that cleavage indeed occurs in endosomes, we performed control experiments with both nonredox‐responsive PCLV and shell‐free C_SS_LV. In the case of the shell‐free C_SS_LV‐containing NBD–PC, only a very limited amount of aggregated structures residing outside of the cells, but no significant cellular uptake is observed (**Figure** **3**). Most likely this is due to inefficient endocytosis and/or compromised stability of the C_SS_LV in the absence of a polymer shell. Nonredox‐responsive PCLV harboring NBD–PC, on the other hand_,_ were internalized into endosomes, but the labeled phospholipid remained entrapped and was not transported to ER membranes (Figure 3). Only the fully redox‐responsive polymer‐shelled P_SS_C_SS_LV containers were efficiently internalized and released their fluorophore‐PC cargo into the cytosol. This indicates that the polymer shell significantly increases stability and endocytic uptake and that both; a reductively cleavable polymer shell and β‐CD_SS_ core are required for efficient release of labeled phospholipids into the cytosol (Figure 3). Thus, P_SS_C_SS_LV represent an alternative cellular uptake and cargo delivery system exploiting redox sensitivity similar to disulfide‐based and thus reduction‐sensitive polymersomes described recently.^28^, ^29^, ^30^, ^31^, ^32^

**Figure 3:**
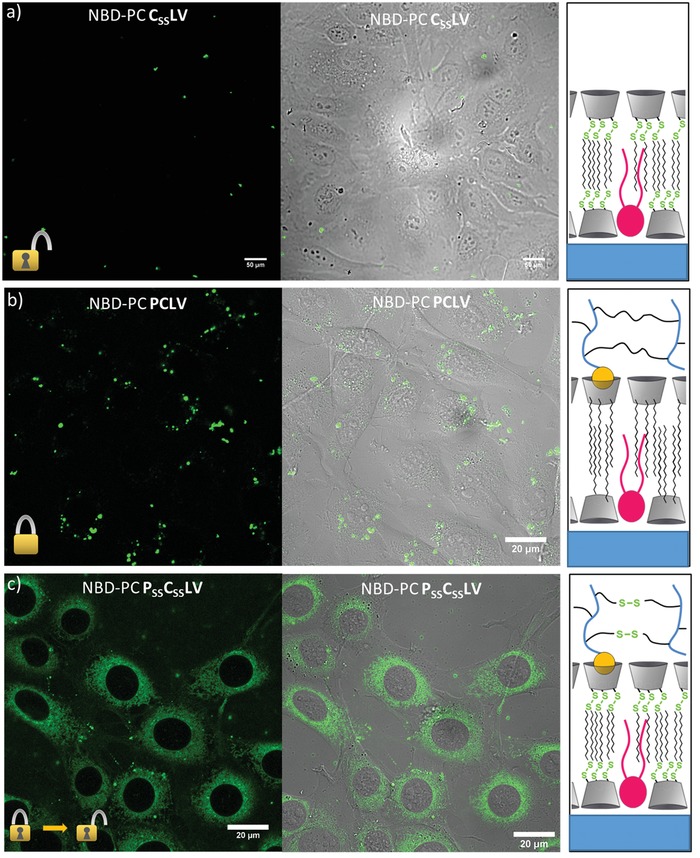
Uptake and endosomal escape of NBD–PC incorporated into different types of nanocontainers. Three different types of containers were analyzed for their capacity to deliver labeled PC into the cytosol of cultivated HUVEC. a) “Open” containers, C_SS_LV, consisting of a redox‐sensitive β‐CD_SS_ core without a polymer shell. Small aggregates visible on top of the HUVEC layer most likely represent noninternalized containers. b) “Closed” containers, PCLV, containing only nonredox‐responsive units. These are taken up into punctuate structures but the labeled PC is not released into the cytosol. c) Redox‐sensitive containers, P_SS_C_SS_LV, with a redox‐responsive core and a redox‐responsive shell, are internalized and the labeled phospholipids are released into the cytosol.

We next examined the cellular delivery of other fluorescently labeled phospholipids incorporated into the redox‐responsive P_SS_C_SS_LV containers. NBD–PA‐containing P_SS_C_SS_LV were also efficiently internalized into endosomes from where the NBD–PA was released into the cytosol accumulating in structures at least in part representing ER membranes. Control experiments with redox‐stable PCLV containers again showed that the cytosolic transport of NBD–PA depended on the presence of a reductively cleavable disulfide bond both, in the β‐CD_SS_ core and in the polymer shell (Figure S10, Supporting Information).

Phosphoinositides (PI) represent another class of negatively charged phospholipids characterized by a highly hydrophilic headgroup that is subject to phosphorylation at different positions. To also analyze this subgroup of phospholipids, we assessed the P_SS_C_SS_LV‐mediated uptake of different PIs. TF–PI‐containing P_SS_C_SS_LV were again efficiently internalized into the endosomal structures. However, at 2 h post delivery only a relatively small portion of the labeled PI appeared in the cytosol, whereas a noticeable signal of TF–PI remained associated with RhB–dextran labeled endosomes. Only prolonged incubation times resulted in substantial cytosolic delivery (Figure S11, Supporting Information). Endosomal retention is even more pronounced in case of the dual phosphorylated PI(4,5)P_2_. When delivered in P_SS_C_SS_LV, TF–PI(4,5)P_2_ remained in RhB–dextran positive endosomes for extended period of time, and even after 24 h no significant release to the cytosol was observed. Since the redox‐induced disassembly of the P_SS_C_SS_LV within endosomes is most likely not dependent on the encapsulated amphiphilic cargo, the difference in cytosolic release kinetics between PC and PA on one hand and phosphoinositide derivatives on the other hand is most likely due to the different head groups. Although the mechanism that triggers the release of the P_SS_C_SS_LV delivered phospholipids from endosomes is not known, the different kinetics observed here argue for an important role of the head group, possibly by affecting a translocation from the luminal to the cytoplasmic leaflet of the endosomal bilayer. It should be noted that after release of the fluorescently labeled lipid from the P_SS_C_SS_LV a further biosynthetic modification of the lipid can occur, both, in endosomes and even more likely at the ER. However, the significant head group differences observed here indicate that major processes such as a rapid degradation of the lipid are unlikely to occur.

In conclusion, we synthesized polymer‐shelled nanocontainers based on redox‐responsive cyclodextrin amphiphiles which were able to deliver different types of fluorescently labeled phospholipids (PC, PA, and PI) to intracellular membrane compartments (endosomes, ER). Mixtures of reductively degradable cyclodextrin amphiphiles and different phospholipids formed liposome‐like cyclodextrin vesicles (C_SS_LV) with a homogeneous distribution of each lipid. C_SS_LV could be encapsulated in a redox‐sensitive polymer shell, and the resulting P_SS_C_SS_LV showed a high stability that was regulated by the redox state of the environment. P_SS_C_SS_LV were efficiently internalized into endosomes of primary human endothelial cells where the incorporated amphiphilic cargo was released via reductive disintegration of the P_SS_C_SS_LV. We also showed that P_SS_C_SS_LV represent a novel tool to deliver fluorescently labeled phospholipids into cells and that the endosomal escape of these externally delivered phospholipids depends on the type of head group. Thus, P_SS_C_SS_LV represent a highly efficient transport system for the delivery of lipid‐like amphiphilic cargo into the intracellular environment.

## Experimental Section

Methods including DLS, FRET, TEM, and microscopic imaging as well as synthesis of C_SS_LV and P_SS_C_SS_LV are described in detail in the Supporting Information.

## Conflict of Interest

The authors declare no conflict of interest.

## Supporting information

Supporting Information
