# Supplementary material for: Controlled Cellular Delivery of Amphiphilic Cargo by Redox‐Responsive Nanocontainers
Source: Adv Sci (Weinh). 2019 Oct 24;6(24):1901935. doi: 10.1002/advs.201901935 (PMC6918114; doi:10.1002/advs.201901935)
Supplement: Supplementary file 1 — Supporting Information [file ADVS-6-1901935-s001.pdf]

Copyright WILEY-VCH Verlag GmbH & Co. KGaA, 69469 Weinheim, Germany, 2019.

ADVANCED SCIENCE
Open Access

Supporting Information

for *Adv. Sci.,* DOI: 10.1002/advs.201901935

Controlled Cellular Delivery of Amphiphilic Cargo by Redox-Responsive Nanocontainers

Wilke C.de Vries, Sergej Kudruk, David Grill, Maximilian Niehues, Anna Livia Linard Matos, Maren Wissing, Armido Studer, Volker Gerke, and Bart Jan Ravoo**

Supporting Information

Controlled Cellular Delivery of Amphiphilic Cargo by Redox-Responsive Nanocontainers

Wilke C. de Vries [†+], *Sergej Kudruk* [‡+], *David Grill* [‡], *Maximilian Niehues* [†], *Anna Livia Linard Matos* [‡], *Maren Wissing* [†], *Armido Studer* [†], *Volker Gerke* [‡*] *and Bart Jan Ravoo* [†*]

[†] Dr. W. C. de Vries, M. Niehues, M. Wissing, Prof. Dr. A. Studer, Prof. Dr. B.J. Ravoo
Center for Soft Nanoscience and Organic Chemistry Institute, Westfälische Wilhelms Universität Münster, Busso Peus Straße 10, 48149, Germany
E-mail: b.j.ravoo@uni-muenster.de

[‡] S. Kudruk, Dr. D. Grill, A. L. L. Matos, Prof. Dr. V. Gerke
Institute of Medical Biochemistry, Center for Molecular Biology of Inflammation, Westfälische Wilhelms Universität Münster, Von-Esmarch-Str. 56, 48149, Germany
E-mail: gerke@uni-muenster.de

[+] Equal contribution
[*] Corresponding authors

Instrumentation

^{1}H-NMR and ^{13}C-NMR spectra were recorded on an *DPX 300* (*Bruker*), Avance II 300 (*Bruker*), *Avance II 400 (Bruker)* or a DD2 600 instrument (*Agilent*). Chemical shifts *δ* in ppm are referenced to the solvent residual peak. **IR spectra** were recorded on a 3100 FT-IR instrument (*Varian*) equipped with a MKII Golden Gate Single Reflection ATR unit (*Varian*). Signals were given in wavenumbers ν (cm^{-1}). Intensities were abbreviated with (*s*) strong, (*m*) medium, (*w*) weak and (*br*) broad. **HRMS (ESI)** was performed using a MicroTOF ESI (*Bruker*) and an Orbitrap LTQ XL (*Thermo Scientific*). **Gel Permeation Chromatography (GPC)** was carried out with extra pure THF as eluent at a flow rate of 1.0 mL min^{-1} at 25°C on a system consisting of a PSS SECurity GPC (*Polymer Standards Service*), a set of two PLgel 5µm MIXED-C columns (300×7.5mm, *Agilent Technologies*) plus a guard column. Data were analyzed with PSS WinGPC Compact software (version.7.20, *Polymer Standards Service*) based upon calibration curves of poly(methylmethacrylate) standards (*Varian*) with peak molecular weights ranging from 1660 to 1000000 g mol^{-1}. **Dynamic light scattering (DLS) and *ζ*-potential** measurements were carried out on a Nano ZS Zetasizer (*Malvern Instruments*) at 25 °C and samples were prepared in disposable 1 mL semi-micro PMMA cuvettes (*BRAND*) or in disposable DTS 1070 capillary cells (*Malvern Instruments*). Data analysis was performed with Zetasizer Software Version 7.12 (*Malvern Instruments*) and OriginPro 9.6.0172 (*Origin*). **Transmission electron microscopy (TEM)** was performed using a Titan Themis G3 300 TEM (*FEI*) operating at 300 kV. Sample preparation was performed by incubation of a glow-discharged carbon-coated copper grid (S162, *Plano GmbH*) with 5 µL of the nanocontainer solution for 1 min and gentle blotting with filter paper. The sample was stained with 5 µL of 0.5% (w/w) aqueous phosphotungstic acid for 30 s and again gently blotted with filter paper. TEM data were analyzed with TEM imaging & Analysis version 4.15 (FEI)and ImageJ version 1.52h (*National Institutes of Health*, USA, Java 1.8.0_66). **Fluorescence measurements and light scattering measurements at *λ* = 400 nm** were performed with a FP 8500 spectrofluorometer (*JASCO*) at 25 °C. Samples for spectroscopic measurements were prepared in disposable 1 mL semi-micro PMMA cuvettes (*BRAND*) or quartz glass cuvettes (*Hellma*) and data analysis emplpoyed Spectra Manager Version 2.14.06 (*JASCO*) and OriginPro 9.6.0172 (*Origin*). **Live-cell imaging and fluorescence microscopy** was performed with a LSM 780 confocal laser scanning microscope (CLSM, *Carl Zeiss*) equipped with the objective lense Plan-Apochromat x 63/1.4 oil differential interference contrast (*Carl Zeiss*) and an Argon-Ion laser (*LASOS*) in a 37 °C environment with5% CO_2. Data were processed with ImageJ version 1.52h (*National Institutes of Health*, USA, Java 1.8.0_66).

Materials

All chemicals were purchased from *Sigma Aldrich*, *Merck*, *VWR*, *Carbolution* or *TCI* and used as delivered unless otherwise stated. 1-palmitoyl-2-oleoyl-*sn*-glycero-3-phospho-(1'-*rac*-glycerol) (sodium salt) (POPG), 1,2-dioleoyl-*sn*-glycero-3-phosphate (sodium salt) (DOPA), 1,2-dioleoyl-*sn*-glycero-3-phosphocholine (DOPC), 1-palmitoyl-2-(dipyrrometheneboron difluoride)undecanoyl-*sn*-glycero-3-phosphocholine (TopFluor® PC), 1-palmitoyl-2-{12-[(7-nitro-2-1,3-benzoxadiazol-4-yl)amino]dodecanoyl}-*sn*-glycero-3-phosphocholine (NBD-PC), 1-oleoyl-2-{12-[(7-nitro-2-1,3-benzoxadiazol-4-yl)amino]dodecanoyl}-*sn*-glycero-3-phosphate (ammonium salt) (NBD PA), 1-oleoyl-2-{6-[4-(dipyrrometheneboron difluoride)butanoyl]amino}hexanoyl-*sn*-glycero-3-phosphoinositol-4,5-bisphosphate (ammonium salt) (TopFluor® PI(4,5)P_2), 1-oleoyl-2-{6-[4-(dipyrrometheneboron difluoride)butanoyl]amino}hexanoyl-*sn*-glycero-3-phosphoinositol (ammonium salt) (TopFluor® PI), 1,2-dioleoyl-*sn*-glycero-3-phosphoethanolamine-*N*-(7-nitro-2-1,3-benzoxadiazol-4-yl) (ammonium salt) (NBD PE) and 1,2-dioleoyl-*sn*-glycero-3-phosphoethanolamine-*N*-(lissamine rhodamine B sulfonyl) (ammonium salt) (LissRhod PE) were purchased from Avanti Polar Lipids. Dulbeccos phosphate buffered saline buffered saline (DPBS, 1.47 mM KH_2PO_4, 7.67 mM Na_2HPO_4, 136.9 mM NaCl, 2.7 mM KCl (all *Sigma Aldrich*), pH 7.4) was prepared using ultrapure water with a resistance higher than 18 MΩ. For cell experiments sterile filtered DPBS with the same composition was purchased from *Sigma Aldrich*.

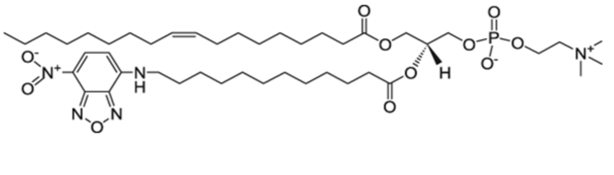

NBD PC

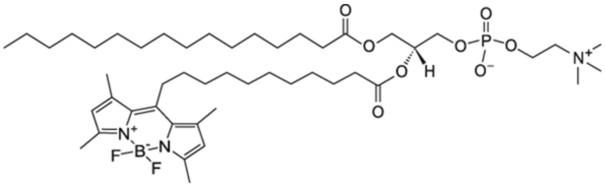

TopFluor® PC

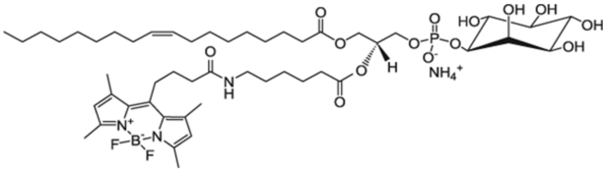

TopFluor® PI

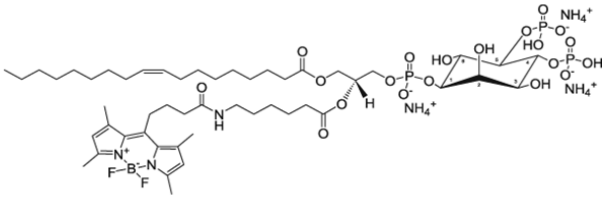

TopFluor® PI(4,5)P$_2$

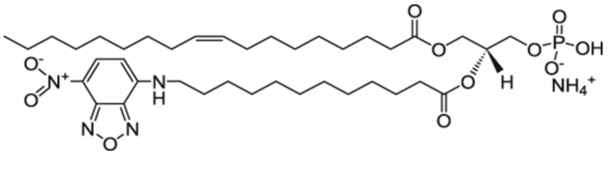

NBD PA

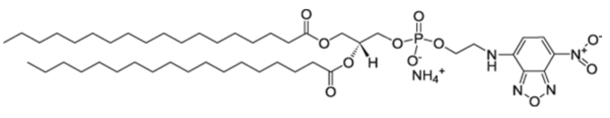

NBD PE

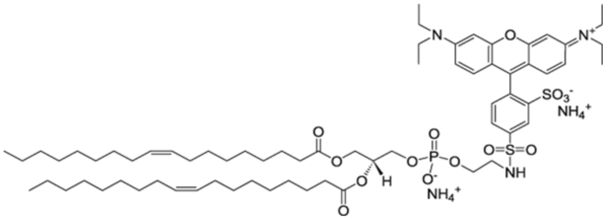

Liss Rhod PE

Scheme S1. Chemical structures of the fluorescently labeled lipids: 1-palmitoyl-2-(dipyrrometheneboron difluoride)undecanoyl-*sn*-glycero-3-phosphocholine (TopFluor® PC), 1-palmitoyl-2-{12-[(7-nitro-2-1,3-benzoxadiazol-4-yl)amino]dodecanoyl}-*sn*-glycero-3-phosphocholine (NBD-PC), 1-oleoyl-2-{12-[(7-nitro-2-1,3-benzoxadiazol-4-yl)amino]dodecanoyl}-*sn*-glycero-3-phosphate (ammonium salt) (NBD PA), 1-oleoyl-2-{6-[4-(dipyrrometheneboron difluoride)butanoyl]amino}hexanoyl-*sn*-glycero-3-phosphoinositol-4,5-bisphosphate (ammonium salt) (TopFluor® PI(4,5)P$_2$), 1-oleoyl-2-{6-[4-(dipyrrometheneboron difluoride)butanoyl]amino}hexanoyl-*sn*-glycero-3-phosphoinositol (ammonium salt) (TopFluor® PI), 1,2-dioleoyl-*sn*-glycero-3-phosphoethanolamine-*N*-(7-nitro-2-1,3-benzoxadiazol-4-yl) (ammonium salt) (NBD PE) and 1,2-dioleoyl-*sn*-glycero-3-phosphoethanolamine-*N*-(lissamine rhodamine B sulfonyl) (ammonium salt) (LissRhod PE).

General Procedures

Preparation of mixed cyclodextrin lipid vesicles (C_{SS}LV): Unilamellar bilayer vesicles of amphiphilic cyclodextrin derivatives and phospholipids were prepared by hydration of a thin film and subsequent extrusion. Briefly, amphiphile stock solutions in chloroform or chloroform/methanol mixtures were mixed in a round bottom flask at a molar ratio of 50 mol% redox-responsive amphiphilic β-cyclodextrin (β-CD_{SS}), 25 mol% POPG, 20 mol% DOPA and 5 mol% DOPC. If indicated the phospholipids were partially replaced by fluorescent analogues (Table S1) and for a redox-stable vesicle template β-CD_{SS} was replaced by redox-stable amphiphilic β-cyclodextrin[1,2]. The solvent was evaporated in a stream of argon to obtain a thin film and residual solvent was removed under high vacuum. The film was hydrated by addition of DPBS to yield a total amphiphile concentration of 100 µM. Upon vigorous stirring for at least 2 h this solution was vortexed, subjected to 10 freeze-thaw cycles and repetitively passed through a polycarbonate membrane with 100 nm pore size (*AVESTIN*) in a Liposofast manual extruder (*AVESTIN*) to yield CLV.

Table S1. Molar fractions of amphiphiles for the preparation of C_{SS}LV templates with different labelled cargo lipids.

Cargo	*without*	*TopFluor® or NBD PC*	*TopFluor® or NBD PA*	*TopFluor® PI(4,5)P₂*
amphiphilic β-CD_{SS}	*50%*	*50%*	*50%*	*50%*
DOPG	*25%*	*25%*	*25%*	*25%*
DOPA	*20%*	*20%*	*15%*	*20%*
DOPC	*5%*	*-*	*5%*	*4%*
TopFluor® or NBD PC	*-*	*5%*	*-*	*-*
TopFluor® or NBD PA	*-*	*-*	*5%*	*-*
TopFluor® PI(4,5)P_2	*-*	*-*	*-*	*1%*

Cargo	*TopFluor® PI*	*NBD PE*	*Liss Rhod PE*
amphiphilic β-CD_{SS}	*50%*	*50%*	*50%*
DOPG	*25%*	*25%*	*25%*
DOPA	*20%*	*20%*	*20%*
DOPC	*4%*	*3%*	*3%*
TopFluor® PI	*1%*	*-*	*-*
NBD PE	*-*	*2%*	*-*
Liss Rhod PE	*-*	*-*	*2%*

Preparation of polymer shelled cylcodextrin/lipid vesicles ($P_{SS}C_{SS}$LV): 20.0 µM Ad-PAA was added to a buffered solution of C_{SS}LV (100 µM total amphiphile concentration in DPBS)

and the pH was readjusted to pH 7.4 by addition of NaOH to obtain polymer decorated cyclodextrin/lipid vesicles ($P_{COOH}C_{SS}LV$). For crosslinking of the polymer shell 33.0 mM 1-ethyl-3-(3-dimethylaminopropyl)carbodiimide hydrochloride (EDC·HCl, *Sigma Aldrich*, corresponding to approximately 3.0 eq. of total carboxylic acid groups) was added. After 20 min, 2.75 mM cystamine (*Sigma Aldrich*, corresponding to approximately 0.25 eq. of total carboxylic acid groups) for a redox-responsive polymer shell or 2.75 mM 2,2′-(ethylenedioxy)bis(ethyleneamine) (*Sigma Aldrich*) for a redox-stable polymer shell were added. The colloid was stirred slowly overnight and the byproducts were removed by dialysis (*Spectra/Por* regenerated cellulose (RC) dialysis membranes, MWCO 1 kDa, *Spectrum Laboratories*) against DPBS (3 × buffer exchange within 24 h) to yield $P_{SS}C_{SS}LV$.

Preparation of giant unilamellar vesicles (GUV): GUV were prepared by gentle swelling on agarose following a procedure modified from Horger et al (2009).[3] Approximately 70 µL of a preheated 2.5% (w/v) solution of ultra-low gelling agarose (*Sigma Aldrich*) in ultrapure water were dropped on a glass slide placed on a hot plate and allowed to dry at 100 °C, followed by drying under high vacuum at room temperature for 1 h. 20 µL of lipid mixtures (2 mM total amphiphile concentration in chloroform or chloroform/methanol mixtures, lipid composition see Table S1) were added dropwise to the agarose film and dried in a stream of argon. Residual solvent was removed under high vacuum. Lipid films were equilibrated in 300µL of 1 mM 4-(2-Hydroxyethyl)piperazine-1-ethanesulfonic acid (HEPES, pH 7.5) buffer for 2 h resulting in the formation of GUV. Prior to microscopy, the GUV solution was carefully transferred onto a clean glass slide and diluted by addition of 200 µL of 1 mM HEPES buffer (pH 7.5). If indicated, 100 nM of adamantly-functionalized rhodamine B[4] (Ad-TEG-RhB) or unfunctionalized rhodamine B were added from concentrated stock solutions prior to fluorescence microscopy imaging.

Disintegration assay monitoring scattered light at 400 nm or Förster resonance energy transfer (FRET): Desintegration of $P_{SS}C_{SS}LV$ was monitored as the decrease of light scattered by the solution of $P_{SS}C_{SS}LV$ corresponding to a total lipid concentration of 25 µM. Dithiothreitol (DTT, *Carbolution*) was added from a concentrated stock solution at $t = 0$ and the intensity of scattered light was measured by using a fluorescence spectrometer ($\lambda_{ex} = \lambda_{em} = 400$ nm). Disintegration of $P_{SS}C_{SS}LV$ was also monitored by recording fluorescence resonance energy transfer (FRET) between *N*-(7-nitro-2,1,3-benzoxadiazol-4-yl), the energy donor, and rhodamine as energy acceptor. The FRET assay was performed essentially as described by Pagano and coworkers.[5] Briefly, DTT was added from a concentrated stock solution at $t = 0$ to a 1:1 mixture of $P_{SS}C_{SS}LV$ (total lipid concentration of 25 µM) either loaded with NBD-PE or Liss Rhod-PE (see Table S1 for lipid composition) and time-dependent fluorescence spectra were recorded. At this concentration scattering contribution to fluorescence was negligible. As a measure of FRET efficiency, the decrease of fluorescence at $\lambda_{em} = 527$ nm ($\lambda_{ex} = 450$ nm) was monitored.

Cell imaging experiments

Cell culture: Primary human umbilical vein endothelial cells (HUVEC), purchased from *PromoCell* (C-12203), were cultured at 37 °C and 5 % CO_2 for up to 5 passages on CellBIND plates (*Corning*, CLS3296-40EA) in HUVEC mix medium consisting of Endothelial Cell Growth Medium 2 (ECGM2, *PromoCell*, C-22011) and M199 medium containing 10% fetal calf serum (FCS) (*Sigma*, F7524) at a 1:1 ratio. The medium was further supplemented with 30 µg/mL gentamycin (*Sigma*, G1397) and 15 ng/mL amphotericin B (*Biochrom*, A2612). For microscopy, HUVEC were cultured in µ-slide 2 well glass bottom (*ibidi*, 80287) pre-coated with collagen (type I).

Uptake: For analyzing the time dependent uptake, freshly prepared $P_{SS}C_{SS}LV$ were dialyzed against M199, diluted 1:3 (end concentration 150 µg/mL) in serum free high glucose DMEM and added to HUVEC for the times indicated in a live cell experimental setup (37 °C and 5% CO_2). For all other uptake experiments, the freshly dialyzed $P_{SS}C_{SS}LV$ were diluted to a final concentration of 150 µg/mL in M199 and incubated with a HUVEC cell layer for 120 min at 37°C and 5% CO_2. After washing 5 times with M199, cell growth was continued in a 1:1 mixture of ECGM2 and M199 with 10% FCS.

Costaining with ER/Endosome marker: For colocalization experiments, ER-Tracker^{TM} Red (BODIPY^{TM} TR Glibenclamide) and Dextran, Texas Red 70000 MW (Thermo Fisher Scientific) (dissolved in DMSO to 1 mM stock solution) were used. The markers were diluted to a 1 µM concentration and were applied to the cells together with the PCLV for 2 h.

Live Cell Microscopy: Live-cell imaging employed a LSM 780 confocal laser scanning microscope (CLSM, *Carl Zeiss*) equipped with the objective lense Plan-Apochromat x 63/1.4 oil and differential interference contrast objective lenses (*Carl Zeiss*) in a 37°C environment with 5 % CO_2. Data were processed using Fiji (*Nat. Methods*, **2012**, *9*, 676).

Synthesis

General: All reactions were carried out in heat-gun-dried glassware under argon atmosphere and were performed by using standard *Schlenk* techniques. Thin layer chromatography was carried out on *Merck* silica gel 60 F254 plates; detection by UV or dipping into a solution of $KMnO_4$ (1.5 g), $NaHCO_3$ (5.0 g) in H_2O (400 mL) followed by heating. Flash chromatography (FC) was carried out on *Merck* silica gel 60 (40 – 63 µm) at an argon pressure of 0-0.5 bar. Adamantanyl initiator/regulator **1**[6], redox-stable amphiphilic β-cyclodextrin[1,2], redox-cleavable amphiphilic β-cyclodextrin[7] and adamantyl-functionalized rhodamine B[4] (Ad-RhB) were prepared according to previously reported procedures.

Synthesis of adamantyl terminated poly(*acrylic acid*) (Ad-PAA)

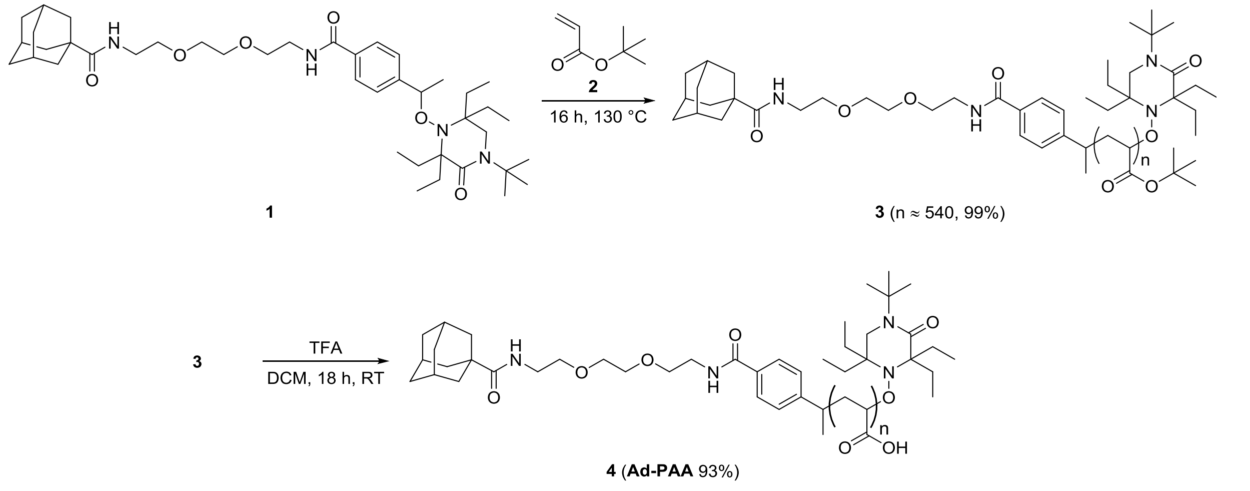

Scheme S2. Synthesis of **Ad-PAA** according to a procedure modified from *Ravoo et al.*[6] starting from adamantyl-initiator **1.**

Adamantyl terminated poly(*tert*-butyl acrylate)

As described by Ravoo *et al.*[6] a heatgun-dried Schlenk tube was charged with adamantly-initiator/regulator **1** (7.2 mg, 10 µmol, 1.0 equiv) and *tert*-butyl acrylate **2** (0.58 mL, 4.0 mmol, 400 equiv). The solution was degassed by conducting three freeze/thaw cycles.[a1] After the mixture was brought to room temperature the tube was sealed and the polymerization was carried out at 130 °C for 16 h. The reaction was cooled to room temperature and transferred to a round bottom flask using DCM. Solvent and residual monomers were removed under reduced pressure and the polymer was dried in vacuo. Conversion was determined gravimetrically. Molecular weight and PDI were determined by GPC at 25 °C against PMMA standards using THF as eluent. The polymer **3** was obtained as a white solid (515 mg, 99%, Mn: 70 500g/mol, DP: 540, PDI: 1.4).

^{1}H NMR (300 MHz, $CDCl_3$, 298K): δ = 2.23 (*br*, 1H), 1.93-1.60 (*br*, 1H), 1.51 (*br*, 1H), 1.44 (*br*, 9H) ppm.

IR (ATR): ν = 2978*w*, 2933*w*, 1723*s*, 1480*w*, 1448*w*, 1393*m*, 1366*s*, 1255*m*, 1037*w*, 918*w*, 845*m*, 733*m*, 648*w* cm^{-1}.

Adamantyl terminated poly(acrylic acid) (Ad-PAA)

Trifluoroacetic acid (2.24 mL, 3.33 g, 29.2 mmol, 10.0 eq.) was added slowly to a solution of the *tert*-butyl protected polymer (374 mg, 2.92 mmol of *tert*-butyl acrylate units, 1.00 eq.) in dry DCM (20 mL). The reaction mixture was stirred at RT for 18 h forming a white precipitate. The solvent was removed under reduced pressure and the polymer was dissolved in ultrapure water while the pH was adjusted to pH 7 by addition of 1 M NaOH. Dialysis (Spectra/Por 7 regenerated cellulose dialysis membranes, MWCO 6−8 kDa) against ultrapure water (3 × exchange of water in 24 h) and subsequent freeze-drying yielded Ad-PAA **4** as a cottony white solid (196 mg, 2.72 mmol, 93%).

^{1}H-NMR (300 MHz, D_2O, 298K): δ = 2.38 (*br*, 1H, CH-2), 2.17 – 1.34 (*br m*, 2 H, CH_2-1) ppm.

IR (ATR): *ν* = 3100*br*, 2940*br*, 2584*br*, 1699*s*, 1451*w*, 1413*w*, 1241*m*, 1163*m*, 850*w*, 799*w* cm^{-1}.

[a1] Freeze-thaw cycles were conducted as follows: Under a strong flow of argon, the tube was placed in a dewar filled with liquid nitrogen. After 5 min, high vacuum was applied for 5 min. Then the tube was removed from the nitrogen bath and maintained under high vacuum for another minute and allowed to warm up to RT under a strong argon flow.

Additional Experimental Data

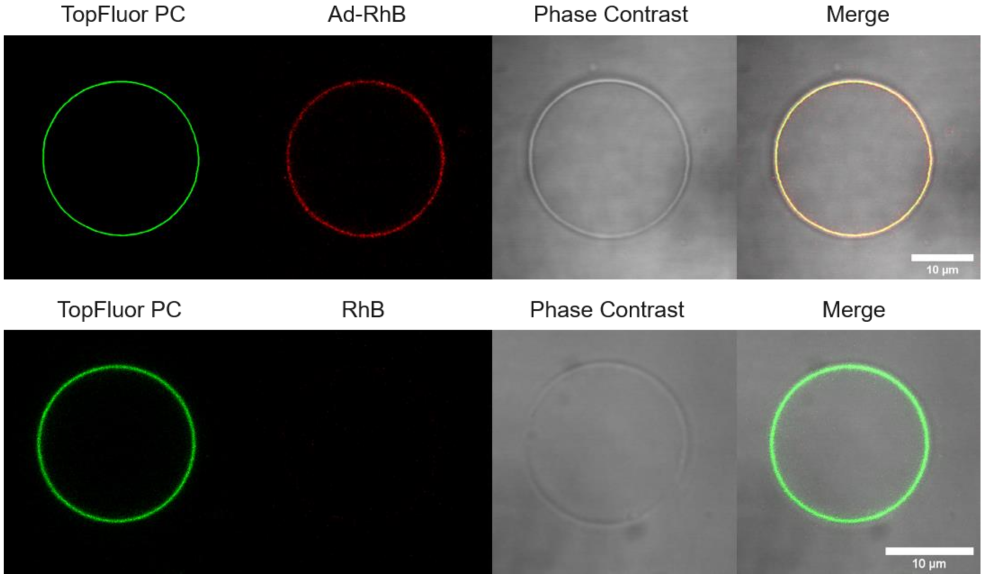

Figure S1. Lipid distribution in giant mixed β-CD_{SS}/lipid vesicles containing TopFluor® PC. Ad-TEG-RhB was added to visualize the CD host units at the vesicle surface (top). A reference experiment with non-functionalized RhB excludes unspecific binding of the rhodamine dye (bottom). Images show confocal sections taken at the equatorial planes of the GUV.

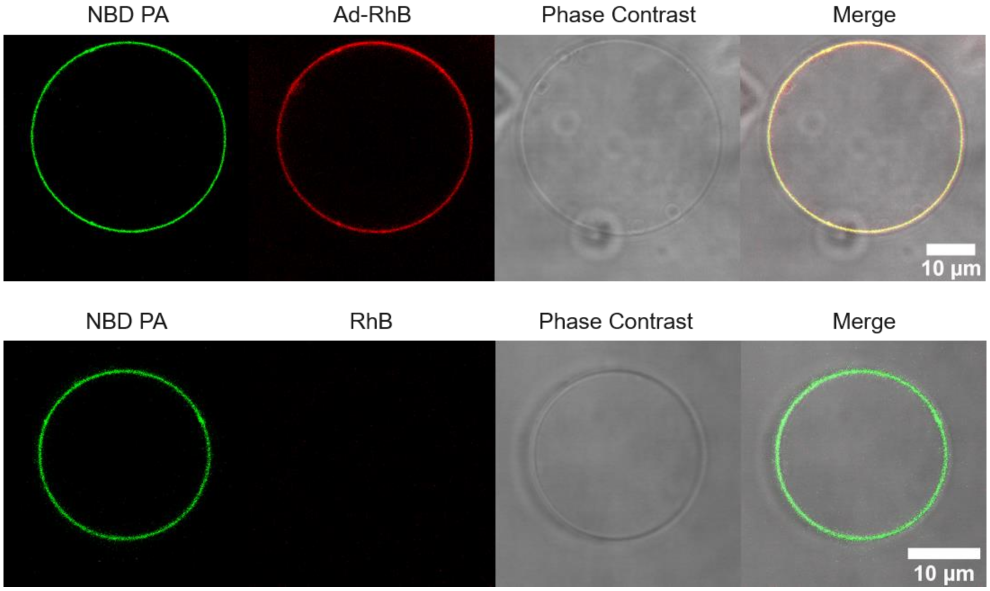

Figure S2. Giant β-CD_{SS}/lipid vesicles incorporating NBD PA as a cargo (see Table S1 for lipid composition). Ad-TEG-RhB was added to visualize the cyclodextrin host units at the vesicle surface (top) and a reference experiment with unfunctionalized RhB was performed to exclude unspecific binding of the rhodamine dye (bottom). Images show confocal sections taken at the equatorial planes of the β-CD_{SS}/lipid vesicles.

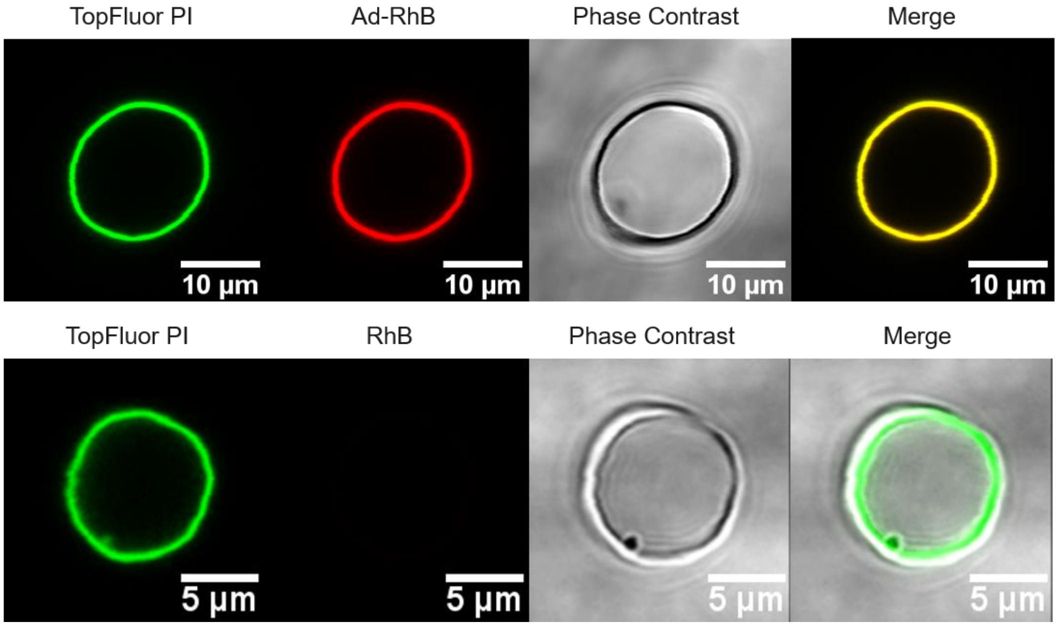

Figure S3. Giant β-CD$_{SS}$/lipid vesicles incorporating TopFluor® PI as a cargo (see Table S1 for lipid composition). Ad-TEG-RhB was added to visualize the cyclodextrin host units at the vesicle surface (top) and a reference experiment with unfunctionalized RhB excludes unspecific binding of the rhodamine dye (bottom). Images show confocal sections taken at the equatorial planes of the GUV.

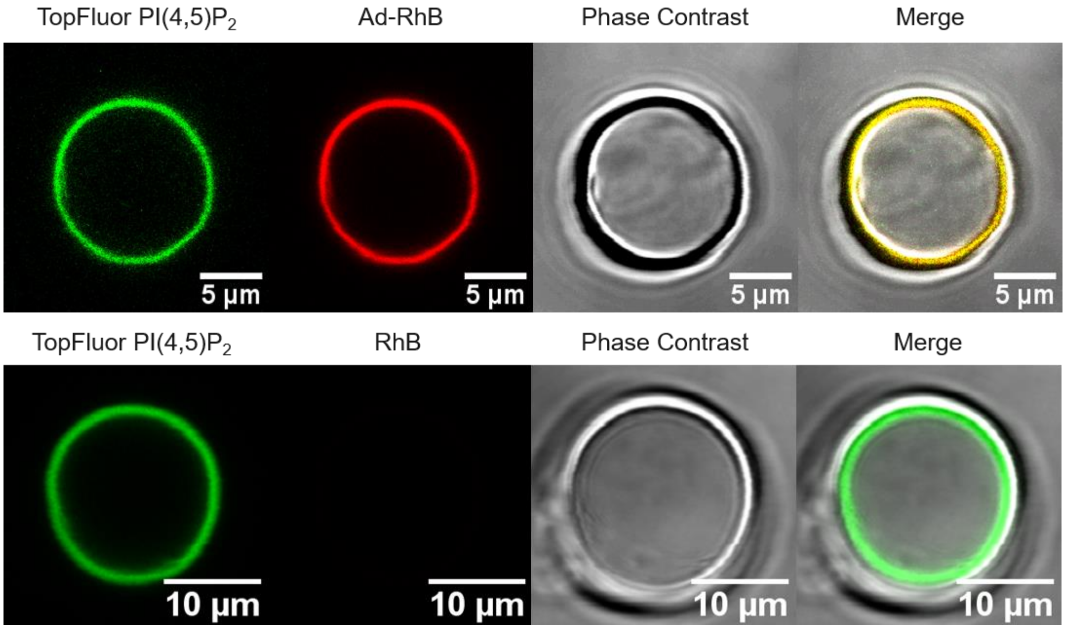

Figure S4. Giant β-CD$_{SS}$/lipid vesicles incorporating TopFluor® PI(4,5)P$_2$ as a cargo (see Table S1 for lipid composition). Ad-TEG-RhB was added to visualize the cyclodextrin host units at the vesicle surface (top) and a reference experiment with unfunctionalized RhB was performed to exclude unspecific binding of the rhodamine dye (bottom). Images show confocal sections taken at the equatorial planes of the GUV.

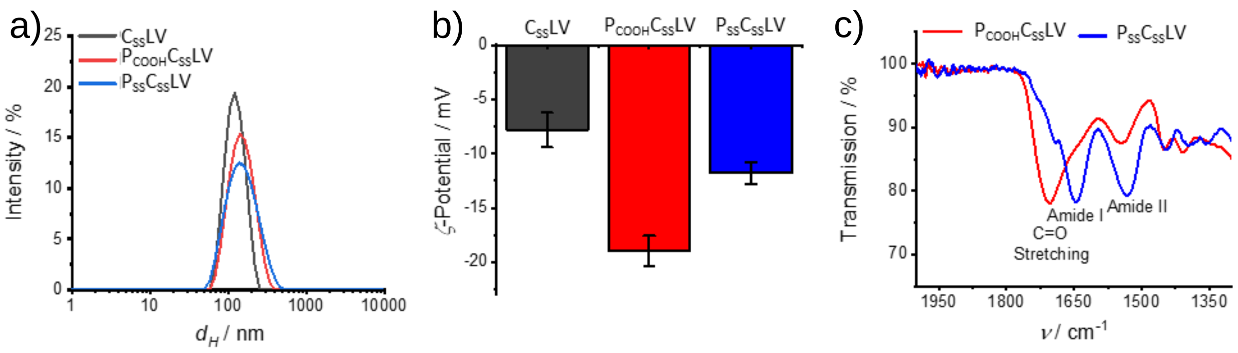

Figure S5. a) Size distribution according to DLS and b) ζ-potential of $C_{SS}LV$, $P_{COOH}C_{SS}LV$ and $P_{SS}C_{SS}LV$ measured in DPBS at pH 7.4. ζ-potential represents average ± SD (N = 5). c) Zoomed-in region showing amide absorption bands of FT-IR spectra of $P_{COOH}C_{SS}LV$ and $P_{SS}C_{SS}LV$ at 1532 cm^{-1} and 1647 cm^{-1}.[8,9]

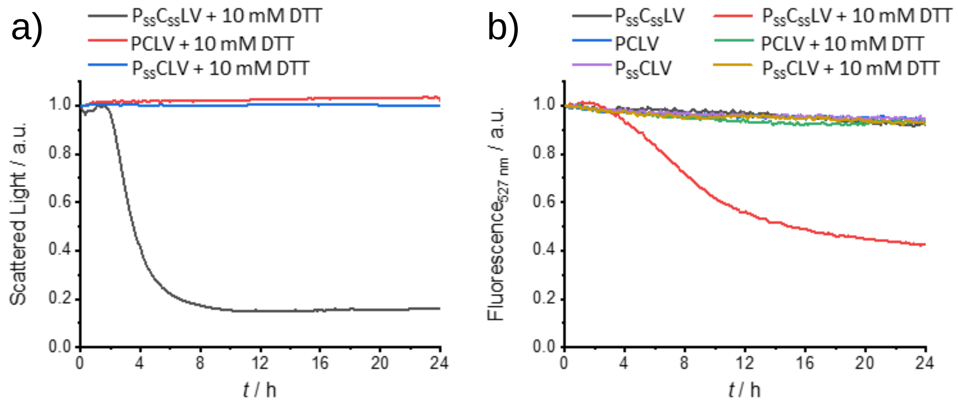

Figure S6. Comparison of the reductive degradation of $P_{SS}C_{SS}LV$ to that of redox-stable PCLV and $P_{SS}CLV$, which only contain a reductively cleavable polymer shell but a redox-stable vesicle template. a) Time-dependent monitoring of the light (λ = 400 nm) scattered from the nanocontainer sample. b) Time-dependent development of a FRET effect quantified by the decreasing fluorescence emission of NBD at λ_{em} = 527 nm (see General Procedure for experimental details).

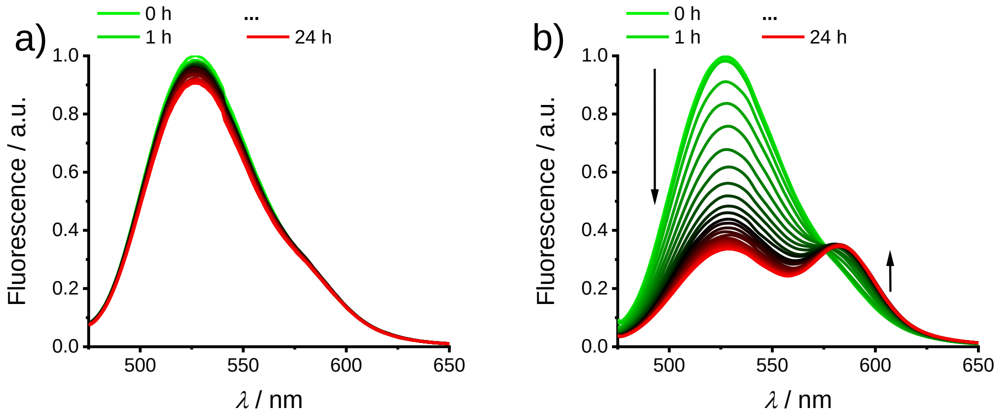

Figure S7. Time-dependent reductive desintegration of $P_{SS}C_{SS}LV$ as revealed by an increasing FRET-effect. a) Fluorescence spectra of a control experiment without addition of DTT. b) Fluorescence spectra of a mixture of $P_{SS}C_{SS}LV$ either loaded with NBD PE or Liss Rhod PE after addition of 20 mM DTT at t = 0 min. Arrows indicate the decrease in donor and increase in acceptor fluorophore emission.

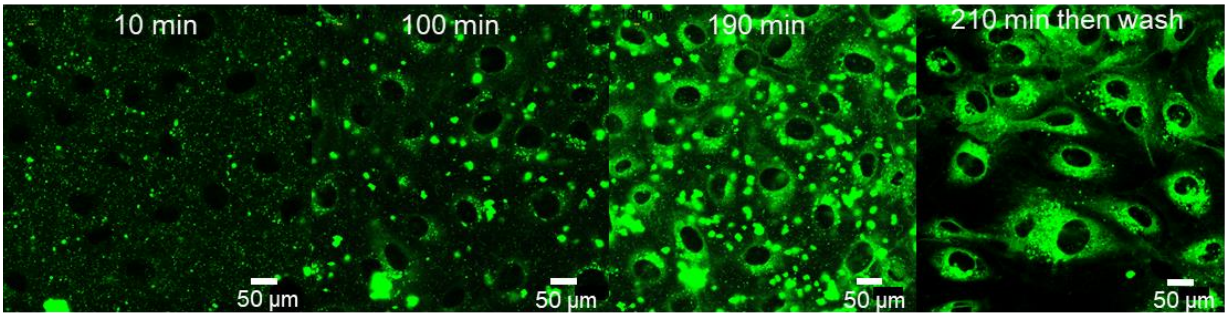

Figure S8. Confocal microscope (CM) images showing the time-dependent release (10 min to 210 min) of TopFluor-PC from $P_{SS}C_{SS}LV$ following uptake into HUVEC. Scale bar, 50 µm.

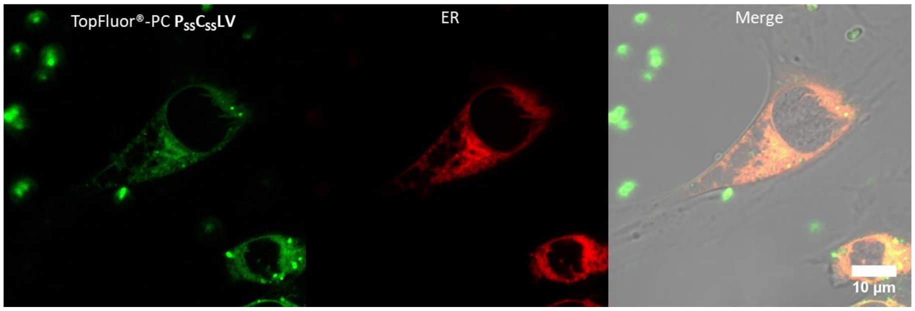

Figure S9. CM images of HUVEC that had been incubated with TopFluor-PC containing $P_{SS}C_{SS}LV$ for 2 h. Note the efficient release into the cytoplasm where the TopFluor label accumulates in structures co-localizing with the ER marker BODIPY™ TR Glibenclamide. The merged image on the right shows the uptake of redox-stable PCLV containing NBD-PA. Here the NBD label remained trapped in punctate structure most likely resembling endosomes.

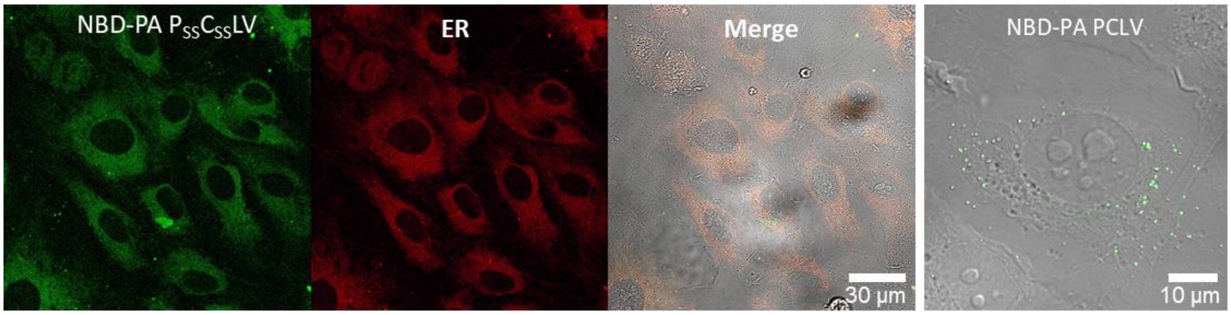

Figure S10. CM images of HUVEC that had been incubated with NBD-PA containing $P_{SS}C_{SS}LV$ for 2 h. Note the efficient release into the cytoplasm where the NBD label accumulates in structures co-localizing with the ER marker BODIPY™ TR Glibenclamide.

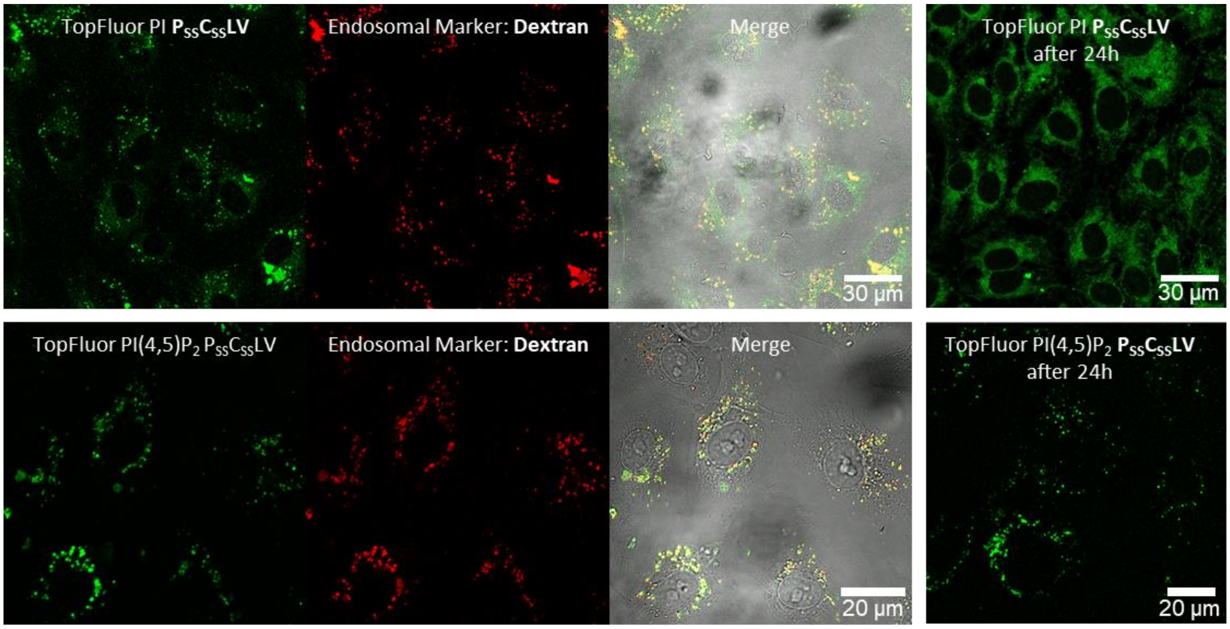

Figure S11. CM images of HUVEC that had been incubated with TopFluor-PI or TopFluor PI(4,5)P_2 containing $P_{SS}C_{SS}LV$ for 2 h (left) or 24 h (right). Note that TopFluor PI is only slowly released into the cytoplasm whereas TopFluor-PI(4,5)P_2 remains in punctate structures most likely resembling endosomes.

References

(1) Ravoo, B. J.; Darcy, R. Cyclodextrin Bilayer Vesicles. *Angew. Chem. Int. Ed.* **2000**, *39*, 4324–4326.

(2) Falvey, P.; Lim, C. W.; Darcy, R.; Revermann, T.; Karst, U.; Giesbers, M.; Marcelis, A. T. M.; Lazar, A.; Coleman, A. W.; Reinhoudt, D. N.; Ravoo, B. J. Bilayer Vesicles of Amphiphilic Cyclodextrins: Host Membranes That Recognize Guest Molecules. *Chem. Eur. J.* **2005**, *11*, 1171–1180.

(3) Horger, K. S.; Estes, D. J.; Capone, R.; Mayer, M. Films of Agarose Enable Rapid Formation of Giant Liposomes in Solutions of Physiologic Ionic Strength. *J. Am. Chem. Soc.* **2009**, *131* (26), 1810–1819.

(4) Kauscher, U.; Stuart, M. C. A.; Drücker, P.; Galla, H. J.; Ravoo, B. J. Incorporation of Amphiphilic Cyclodextrins into Liposomes as Artificial Receptor Units. *Langmuir* **2013**, *29*, 7377–7383.

(5) Struck, D. K.; Hoekstra, D.; Pagano, R. E. Use of Resonance Energy Transfer To Monitor Membrane Fusion. *Biochemistry* **1981**, *20*, 4093–4099.

(6) Samanta, A.; Tesch, M.; Keller, U.; Klingauf, J.; Studer, A.; Ravoo, B. J. Fabrication of Hydrophilic Polymer Nanocontainers by Use of Supramolecular Templates. *J. Am. Chem. Soc.* **2015**, *137*, 1967–1971.

(7) Nolan, D.; Darcy, R.; Ravoo, B. J. Preparation of Vesicles and Nanoparticles of Amphiphilic Cyclodextrins Containing Labile Disulfide Bonds. *Langmuir* **2003**, *19* (10), 4469–4472.

(8) Lee, S.-M.; Chen, H.; Dettmer, C. M.; O'Halloran, T. V.; Nguyen, S. T. Polymer-Caged Lipsome: PH-Responsive Delivery System with High Stability. *J. Am. Chem. Soc.* **2007**, *129*, 15096–15097.

(9) Barth, A. Infrared Spectroscopy of Proteins. *Biochim. Biophys. Acta* **2007**, *1767*, 1073–1101.
